# c-Myc: an emerging participant in heart failure

**DOI:** 10.3389/fcvm.2026.1832539

**Published:** 2026-07-22

**Authors:** Yifeng Wu, Jun Zhang, Ruixin Liu, Wenyue Lin, Wujiao Wang, Yingkun Zhao, Yi Cai, Hong Su, Wenting Sun, Haiyan Zhu, Mingjing Zhao, Peifen Chang

**Affiliations:** 1 Beijing University of Chinese Medicine Affiliated Dongzhimen Hospital, Beijing, China; 2 Beijing University of Chinese Medicine, Beijing, China; 3 Department of Cardiovascular, Ordos Hospital of Traditional Chinese Medicine, Ordos, China; 4 Beijing Bei Zhong Asset Management Co. Ltd, Beijing, China; 5 Guo Weiqin National Famous Traditional Chinese Medicine Inheritance Studio, Beijing, China

**Keywords:** cardiac remodeling, cardiac repair, c-Myc, energy metabolism, heart failure

## Abstract

Heart failure is a significant disease that threatens human life and health globally, yet its pathogenesis remains incompletely understood, posing challenges for targeted treatment. The pleiotropic transcription factor c-Myc participates in the regulation of various biological processes and has been extensively studied in the field of oncology. Recent studies have increasingly indicated that c-Myc is also associated with the development of non-oncological diseases such as heart failure, playing a role in regulating key biological processes including cardiac hypertrophy, cardiac fibrosis, energy metabolism, and cardiac regeneration and repair in heart failure. This article reviews the latest research progress on c-Myc in heart failure, discussing its role in the pathogenesis of heart failure, its clinical significance, its potential as a therapeutic target, and the regulatory effects of traditional Chinese medicine on c-Myc. c-Myc is widely involved in the pathological process of the occurrence and development of HF, but its function is highly diverse. The specific effects of c-Myc vary considerably depending on the pathological context, disease stage, and its expression level. Current research on c-Myc is primarily at the preclinical stage. In order to facilitate the clinical translation of c-Myc as a potential therapeutic target for HF, future research may focus on precise targeting of c-Myc, optimal expression and timing, exploration of its mechanisms, and the potential of traditional Chinese medicine in modulating its effects.

## Graphical abstract

**Figure:**
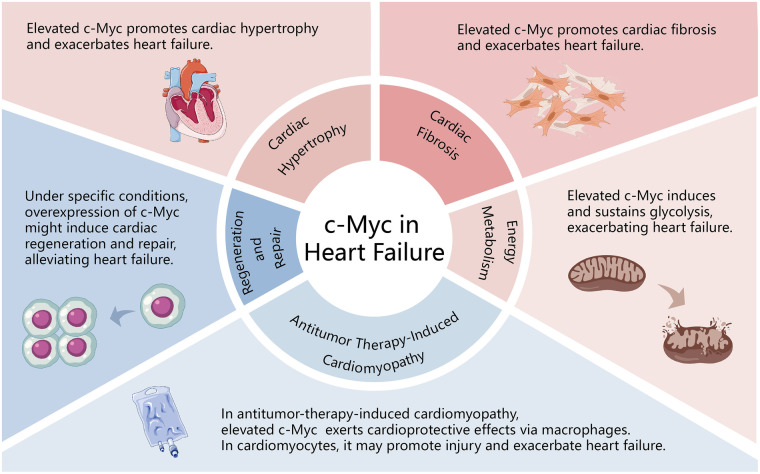
The mechanisms of c-Myc in heart failure.

## 1 Introduction

Heart failure (HF) is a significant disease that threatens human life and health globally. The global prevalence of HF ranges from approximately 1% to 3%, with an estimated 56.2 million patients worldwide (1). As the end-stage manifestation of various cardiovascular diseases, HF has a complex pathogenesis involving multiple biological processes such as cardiac hypertrophy, cardiac fibrosis, immune inflammation, and energy metabolism disorders. These pathological changes collectively lead to impairments in cardiac structure and function, ultimately resulting in the failure of both systolic and diastolic function (2, 3). Therefore, a deeper understanding of the pathogenesis of HF is crucial for developing future targeted treatments.

The *c-Myc* gene encodes a nuclear protein that belongs to the basic helix-loop-helix leucine zipper superfamily. As a pleiotropic master transcription factor, it plays a pivotal role in modulating gene expression programs (4). c-Myc is profoundly involved in numerous core biological processes, including cell cycle progression, ribosome biogenesis, cellular metabolic reprogramming, protein translation, as well as cell differentiation and apoptosis (5). c-Myc is at the crossroads of many growth-promoting signal transduction pathways and constitutes an immediate early response downstream of many ligand-membrane receptor complexes, which underlies its role as a proto-oncogene (6, 7). Given its direct role in promoting tumor initiation and development in numerous human cancers, c-Myc has unsurprisingly emerged as a major molecular target of intense research in cancer biology (8).

However, the functional pleiotropy of c-Myc in cell biology implies that its pathophysiological impact extends far beyond the realm of cancer. In recent years, a growing body of knowledge has illuminated that dysregulated c-Myc expression, encompassing both aberrant activation and functional deficiency, is critically implicated in the pathogenesis of a diverse spectrum of non-oncological diseases (9–11). c-Myc is not merely an oncogene but rather a broad master regulator of cell fate and homeostasis, whose functional networks warrant further in-depth exploration (12). Recent studies have linked c-Myc to the development of HF, demonstrating its involvement in the dysregulation of pivotal biological processes including cardiac hypertrophy, cardiac fibrosis, cardiomyocyte repair and regeneration, energy metabolism, and immune inflammation. This review, therefore, aims to summarize recent advances in understanding the role of c-Myc in HF and to provide a comprehensive overview of its multifaceted functions.

## 2 The structure and biological function of c-Myc

**Figure 1:**
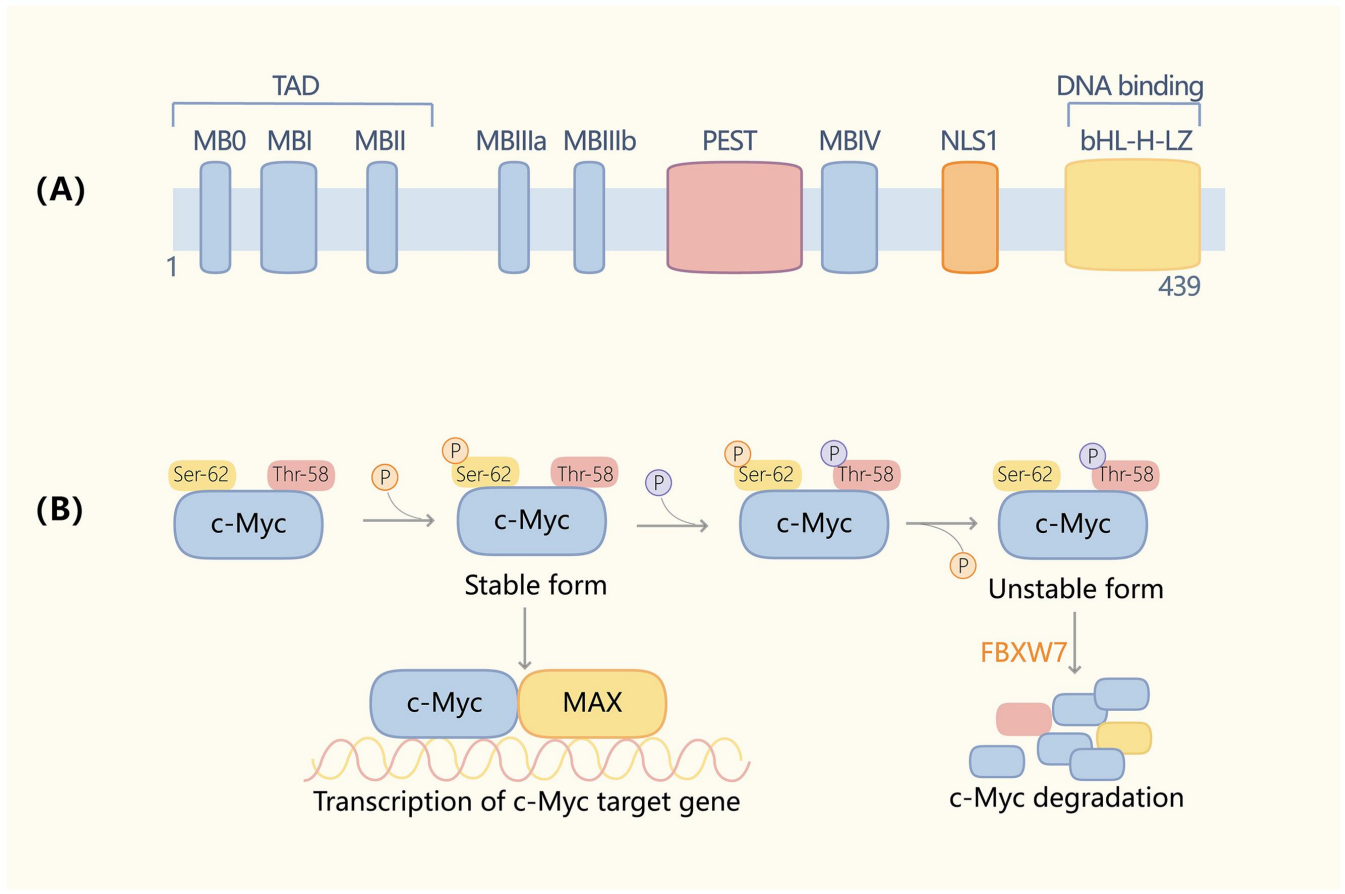
The c-MYC protein **(A)** the c-MYC protein structure with all the main domains annotated; **(B)** the stabilization and degradation mechanism of c-Myc protein. bHL-H-LZ, basic Helix-Loop-Helix Leucine Zipper Domain; FBXW7, F-Box and WD repeat domain-containing 7; MAX, Myc-associated protein X; MB, Myc boxe; NLS, nuclear localization signal; PEST, proline, glutamic acid, serine, and threonine - rich domain; Ser, S erine; TAD, transactivation domain; Thr, threonine.

*Myc* was first identified in 1977 by Duesberg et al. during the isolation and characterization of the avian retrovirus MC29 (13). Subsequently, in 1982, Abrams et al. identified the cellular homolog of c-Myc in the avian genome (14). The human *c-Myc* gene is located at the long arm of chromosome 8, region 2, band 4, sub-band 2, sub-sub-band 1(8q24.21), consisting of three exons interspersed with two intronic regions (15). The canonical translation initiation codon is an ATG codon situated at the beginning of exon 2, which initiates the translation of a 439-amino acid pleiotropic transcription factor (16). Structurally, the c-Myc protein consists of an N-terminal transactivation domain (TAD), a central region, and a C-terminal basic helix-loop-helix (bHLH) domain (17). The TAD structure contains multiple evolutionarily conserved elements known as Myc boxes (MB), including MB0, MBI, and MBII, which interact with a wide array of proteins involved in regulating chromatin remodeling and transcriptional processes (18). The central region consists of the remaining MBIIIa, MBIIIb, and MBIV; a proline, glutamic acid, serine, and threonine - rich domain; and a nuclear localization signal-1 (19). The central region is associated with post-translational modifications and degradation of c-Myc (20). Meanwhile, the C-terminal bHLH domain possesses DNA-binding capability (21, 22). It mediates heterodimerization with Myc-associated protein X (MAX), binding to the regulatory regions of target genes (23). The association between MAX and c-Myc is crucial for activating c-Myc's transcriptional activity (24) (Figure 1 A).

In normal cells, the function and stability of c-Myc are subjected to stringent regulation, and its remarkably short intracellular half-life of approximately 20–30 min (25). This rapid turnover is attributable to its regulation by a variety of post-translational modifications, such as phosphorylation, lactylation, and ubiquitination (26). Within the MBI region of c-Myc reside two evolutionarily conserved phosphorylation sites: threonine 58 (Thr58) and serine 62 (Ser62) (27). Phosphorylation at Ser62 enhances the stability of the c-Myc protein, exerting its transcriptional activity by facilitating its nuclear translocation and heterodimerization with MAX. Intriguingly, phosphorylation at Ser62 primes the protein for subsequent phosphorylation at Thr58, which in turns induces the removal of the phosphate group at S62, leading to a mono-phosphorylated Myc species at Thr58 (28, 29). This unstable state of c-Myc can be specifically recognized by the E3 ubiquitin ligase adapter FBXW7, which mediates its polyubiquitination and targets it for degradation by the 26S proteasome (30, 31). Furthermore, recent research has suggested that under hypoxic conditions, c-Myc can also undergo lactylation on specific lysine residues, a modification that enhances its transcriptional activity and will be discussed in detail in Section 3.4 (32). (Figure 1B)

In tumor cells, the expression of c-Myc is aberrantly elevated under the combined influence of multiple factors. Genomic abnormalities, such as gene amplification and chromosomal translocations, can directly increase c-Myc protein expression (33). Concurrently, dysregulation of upstream signaling pathways also can lead to a marked accumulation of c-Myc, such as Wnt/*β*-catenin, Protein Kinase B (Akt)/Phosphoinositide 3-Kinase (PI3 K), and Extracellular Signal-Regulated Kinase (ERK) signaling pathway (34–37). They can directly activate c-Myc expression or impair its degradation. The extensively activated c-Myc contributes to multiple pathological processes in tumors, including: cell proliferation, metabolic reprogramming, enhanced ribosome and protein biosynthesis, decreased chromosomal stability, aberrant angiogenesis, and upregulation of immune checkpoint inhibitory proteins (38).

In summary, the unique structural and functional characteristics of c-Myc endow it with a critical role in numerous physiological and pathological processes, extensively regulating diverse biological activities related to cell proliferation and metabolism. It follows that the functional impact of c-Myc is not confined to the sphere of oncology as its overexpression, dysregulation, or deficiency can contribute to a range of other diseases. In recent years, accumulating evidence has suggested that c-Myc participates in various pathophysiological alterations in cardiac diseases such as HF, including cardiac hypertrophy, cardiac fibrosis, cardiomyocyte regeneration, and energy metabolism disturbances. The following sections will focus on the mechanisms underlying c-Myc induction in HF and its specific roles in the progression of HF (Table 1).

**Table 1:** c-Myc in heart failure.

Subject	Model	Mechanism	c-Myc’s Effects in Pathological Processes					Reference
			Cardiac hypertrophy	Cardiac fibrosis	Cardiomyocyte proliferation and apoptosis	Energy Metabolism	Immune inflammation	
Sprague-Dawley rat-derived ventricular myocytes	Phenylephrine -induced hypertrophy	During cardiac stress, SIK1 phosphorylated and stabilized HDAC7 protein, and HDAC7 induces c-Myc expression.	Promoting cardiac hypertrophy	-	-	-	-	(46)
C57BL/6J mice	Mule^fl/fl(y)^ mice Myc^fl/fl^ mice Mule^fl/fl(y)^; Myc^fl/fl^ mice	The downregulation of Mule increases the abundance of Myc protein, which in turn downregulates Pgc-1*α* and Pink1.	Promoting cardiac hypertrophy	Promoting cardiac fibrosis	-	Promoting mitochondrial dysfunction	-	(48)
C57BL/6J mice	Ang II infusion via osmotic minipump	EZH2 curbs HUWE1 expression by promoting H3K27me3 modification to enhance c-Myc protein stability, contributing to HF	Promoting cardiac hypertrophy	-	-	-	-	(49)
C57BL/6J mice and H9C2 cell	***In vivo*:** Ang II infusion via osmotic minipump; ***in vitro*:** c-Myc siRNA	Diacerein protects against heart failure through inhibiting MAPKs/c-Myc signaling axis	Promoting cardiac hypertrophy	Promoting cardiac fibrosis	-	-	Promoting inflammation	(50)
Sprague Dawley rats	Coronary artery ligation	Linggui Zhugan decoction inhibits the expression of Wnt/*β*-catenin signaling pathway and c-Myc, and improves cardiac function.	Promoting cardiac hypertrophy	Promoting cardiac fibrosis	-	-	-	(59)
C57BL/6J mice-derived cardiac fibroblasts	Ischemia-reperfusion surgery	Fibronectin polymerization inhibitor pUR4 reduces fibroblast proliferation in heart failure by downregulating the c-Myc signaling axis.	-	Promoting fibroblast proliferation	-	-	-	(62)
C57BL/6 mice-derived cardiac non-myocytes	Transverse aortic constriction surgery	A HF-specific cardiac fibroblasts population contribute to HF via the MYC-CXCL1 pathway,	Promoting cardiac hypertrophy	-	-	-	-	(63)
Drosophila fly, C57BL/6J mice, and mice-derived cardiomyocytes	Treatment with 2-deoxy-D-glucose	SARS-CoV-2 Nsp6 protein promoted the formation of the c-Myc/MAX complex，activated glycolysis, leading to COVID-19-associated heart failure.	-	-	-	Promoting activation of glycolysis and disruption of mitochondrial function	-	(68)
FVB bi-transgenic mice	Cardiac-specific, inducible Myc activation model	The induction of Myc expression in cardiomyocytes leads to progressive ventricular dysfunction and culminates in heart failure.	Promoting cardiac hypertrophy	-	Promoting cardiomyocyte cell cycle reentry	Promoting alterations in mitochondrial function	-	(71)
C57BL/6 × C3H mice	Cardiac-specific, inducible Myc activation model	Activation of Myc provokes cell cycle reentry in postmitotic myocytes, leading to increased nuclei per myocyte and DNA content per nuclei.	Promoting cardiac hypertrophy	-	Promoting multinucleation without cytokinesis	-	-	(78)
Mice and mice-derived cardiomyocytes	LAD ligation in adult mice and apical resection in mice at P7	Transient, cardiomyocyte-specific expression of OSKM extends the regenerative window for postnatal mouse hearts and induces a gene expression program in adult cardiomyocytes that resembles that of fetal cardiomyocytes.	-	-	Promoting mammalian cardiomyocyte cell cycle reentry	-	-	(79)
Mice of a mixed background, and mice-derived cardiomyocytes	LAD ligation	Myc promotes functional and morphological improvements in the heart following acute ischemia and this response correlates with promotion of hyperplasia versus hypertrophy.	-	-	Promoting generation of new binucleated cardiomyocyte	-	-	(80)
C57BL/6J mice and mice-derived cardiomyocytes	LAD ligation	Snhg1 effectively improves cardiac function post-myocardial infarction by forming a positive feedback loop with c-Myc to sustain PI3 K/Akt signaling activation.	Inhibiting cardiac hypertrophy	-	Promoting cardiomyocyte proliferation	-	-	(81)
ICR mice and mice-derived cardiomyocytes	LAD ligation in adult mice and apical resection in mice at P7	NASP transcriptionally upregulates PDGFRβ, subsequently activating the downstream Akt/c-MYC pathway to promote cardiac regeneration and repair after injury.	Inhibiting cardiac hypertrophy	Inhibiting cardiac fibrosis	Promoting cardiomyocyte proliferation	-	-	(85)
ICR mice and mice-derived cardiomyocytes	LAD ligation in adult mice and apical resection in mice at P7	CNBP promotes β-catenin transcription, increases the expression of its downstream target genes cyclin D1 and c-Myc, and ameliorates myocardial injury and cardiac function.	Inhibiting cardiac hypertrophy	Inhibiting cardiac fibrosis	Promoting cardiomyocyte proliferation	-	-	(83)
C57BL/6J mice and mice-derived cardiomyocytes	LAD ligation in adult mice and apical resection in mice at P7	Mydgf improves myocardial injury and cardiac function by activating the c-Myc/FoxM1 pathway.	-	-	Promoting cardiomyocyte proliferation	-	-	(87)
ICR mice and mice-derived cardiomyocytes	LAD ligation in adult mice and apical resection in mice at P7	CircIGF1R inhibits DDX5 degradation, subsequently triggering the β-catenin signaling pathway and leading to transcriptional activation of cyclin D1 and c-Myc, alleviating cardiac dysfunction after injury.	-	Inhibiting cardiac fibrosis	Promoting cardiomyocyte proliferation	-	-	(84)
C57BL/6J mice	DOX administration to induce cardiomyopathy	The SR-A1-c-Myc axis augments the proliferation of cardiac resident reparative macrophages，improving cardiac function in DOX-induced cardiomyopathy.	-	-	-	-	Augmentation of cardiac resident reparative macrophage proliferation	(93)
Sprague-Dawley rats	DOX administration to induce cardiomyopathy	Gen inhibits ERK1/2's phosphorylation, increases the protein levels of STAT3 and c-Myc, improving cardiac function in DOX-induced cardiomyopathy.	-	-	Inhibiting cardiomyocyte cell autophagy and apoptosis	-	-	(94)
C57BL/6 J mice	DOX administration to induce cardiomyopathy	Deubiquitinating effects of OTUB1 on K48- and K63-linked ubiquitination of c-Myc protein are essential for promoting DOX-induced cardiomyopathy.	-	Promoting fibroblast proliferation	Promoting cardiomyocyte apoptosis and oxidative	-	-	(95)

SIKs, salt-inducible kinases; HDAC7, histone deacetylase 7; Pgc-1α, Peroxisome proliferator-activated receptor gamma coactivator 1α; Pink1, Phosphatase and tensin homolog deleted on chromosome ten - induced kinase 1; Ang II, Angiotensin II; EZH2, Enhancer of Zeste Homolog 2; HUWE1, HECT, UBA and WWE Domain Containing 1; H3K27me3, Trimethylation of histone H3 at lysine 27; HF, Heart Failure; MAPK, Mitogen-Activated Protein Kinase; CXCL1, C-X-C Motif Chemokine Ligand 1; P7, postnatal day 7; LAD, left anterior descending coronary artery; OSKM, Oct4, Sox2, Klf4 and c-Myc; Snhg1, lncRNA small nucleolar RNA host gene 1; PI3K, Phosphoinositide 3-Kinase; Akt, Protein Kinase B; ICR, Institute of Cancer Research; NASP, Nuclear Autoantigenic Sperm Protein; PDGFRβ, Platelet-Derived Growth Factor Receptor β; CNBP, cellular nucleic acid binding protein; Mydgf, myeloid-derived growth factor; FoxM1, Forkhead box protein M1; CircIGF1R, circular insulin-like growth factor 1 receptor; DDX5, DEAD-box helicase 5; DOX, Doxorubicin; Gen, Genistein; ERK1/2, extracellular signal-regulated kinase1/2; STAT, signal transducer and activator of transcription; OTUB1, Otubain-1.

## 3 c-Myc in heart failure: detrimental effects

### 3.1 c-Myc is a critical driver of cardiac hypertrophy

**Figure 2:**
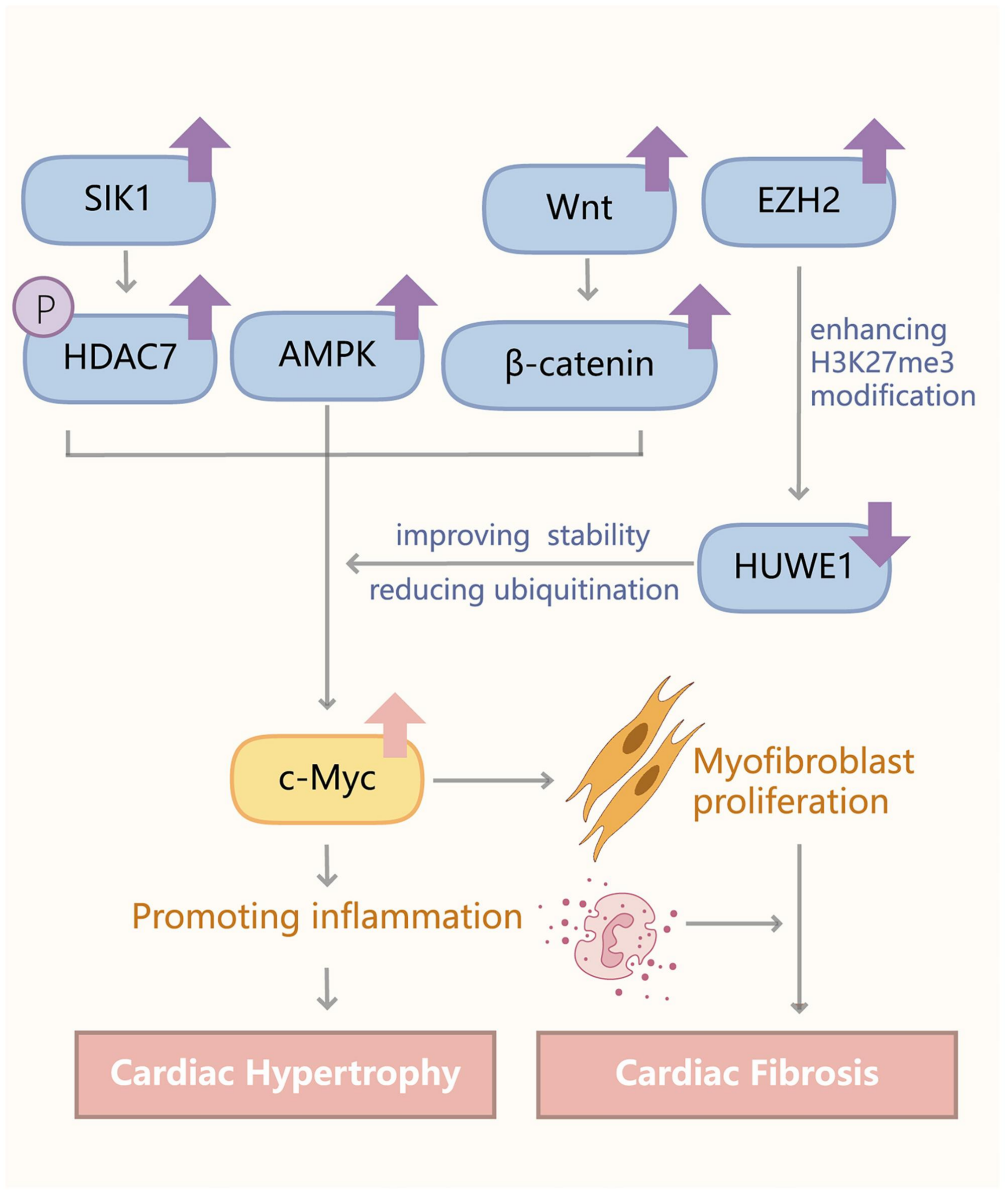
Role of c-Myc in cardiac hypertrophy and fibrosis in heart failure in heart failure cardiomyocytes, signaling pathways including SIK1/HDAC7, Wnt/*β*-catenin, and MAPK upregulate c-Myc expression. EZH2 enhances c-Myc protein stability by suppressing its ubiquitin-mediated degradation, thereby promoting inflammation, cardiac hypertrophy, and cardiac fibrosis. AMPK, adenosine monophosphate-activated protein kinase; EZH2, enhancer of zeste homolog 2; HDAC7, histone deacetylase 7; H3K27me3, histone H3 lysine 27 trimethylation; HUWE1, HECT, UBA and WWE domain containing 1; SIK1, salt-inducible kinase 1.

Cardiac hypertrophy, which persists throughout the onset and progression of HF, serves as an independent predictor of adverse cardiovascular outcomes (39). Cardiac hypertrophy is mainly caused by increased hemodynamic load, broadly categorized either pressure or volume overload. Pressure overload inducing cardiac hypertrophy is characterized by concentric hypertrophy with thickened left ventricular walls, whereas volume overload primarily presents as eccentric hypertrophy with enlarged chamber diameters (40, 41). These structural abnormalities ultimately result in cardiac pump dysfunction. Cardiac hypertrophy primarily relies on the enlargement of individual cardiomyocytes rather than cardiomyocyte proliferation (42). Studies have shown that c-Myc is strongly associated with pathological cardiomyocyte hypertrophy (43). Studies in human myocardial samples have shown that while c-Myc expression is undetectable in normal adult hearts, it is upregulated in myocardial tissues from patients with hypertrophic cardiomyopathy or idiopathic dilated cardiomyopathy. Moreover, the cardiomyocytes exhibit larger cell volume in c-Myc-positive individuals than in c-Myc-negative individuals (44, 45), suggesting that c-Myc contributes to both concentric and eccentric cardiac hypertrophy (Figure 2).

Similar phenomena have been observed in various animal models, consistent with the findings in human tissues. In pressure overload-induced models, c-Myc was activated via signaling pathways such as the upstream salt-inducible kinase 1 (SIK1)/histone deacetylase 7 (HDAC7), resulting in elevated levels of both its transcription and protein expression. While knockdown of c-Myc alleviates transverse aortic constriction-induced cardiomyocyte hypertrophy (46). HECT, UBA, and WWE Domain-Containing 1 (HUWE1), previously known as Mule, is an E3 ubiquitin ligase that targets c-Myc for ubiquitination and degradation (47). In Mule-deficient mice, accumulation of c-Myc was observed, leading to spontaneous cardiac hypertrophy, left ventricular dysfunction, and premature mortality (48). Similarly, in angiotensin II (Ang II)-induced cardiac hypertrophy models, enhancer of zeste homolog 2 (EZH2) stabilized c-Myc protein by inhibiting its ubiquitination-mediated degradation, thereby promoting inflammation and cardiomyocyte hypertrophy. Overexpression of c-Myc exacerbated these pathological alterations (49), whereas c-Myc knockdown suppressed them (50). In summary, as a core regulator of pathological cardiac hypertrophy, c-Myc is activated and stabilized through multiple signaling pathways, driving both concentric and/or eccentric cardiac hypertrophy and ultimately culminating in HF.

### 3.2 c-Myc mediates cardiac fibrosis by regulating cardiomyocytes and fibroblasts

Myocardial fibrosis is a critical pathological process in HF, characterized by excessive accumulation of extracellular matrix and aberrant collagen deposition in cardiac tissue (51). Following myocardial injury induced by pathological stimuli (e.g., ischemia, overload), activated immune cells are recruited to eliminate dead or damaged cardiomyocytes (52). Concurrently, immune cells and damaged cardiomyocytes secrete pro-inflammatory cytokines (53, 54), thus inducing an immune-inflammatory state and activating myofibroblasts (MFs) (55, 56). The hyperactivated MFs disrupt the homeostasis between collagen synthesis and degradation, and promoting abnormal remodeling of the collagen network (57). This process initially serves as a protective measure, in which extracellular matrix proteins provide structural support to the heart. However, excessive fibrosis leads to tissue stiffening, ultimately progressing to ventricular structural abnormalities and impaired systolic and diastolic function (58). Throughout this process, c-Myc participates in the regulation of both cardiomyocyte and fibroblast (Figure 2).

In cardiomyocytes, upregulation of c-Myc promotes cardiac fibrosis, which has been reported across multiple animal models of HF. In HF model induced by transverse aortic constriction, HDAC7, as a pro-hypertensive signaling effector, induced c-Myc upregulation, thereby promoting inflammation and myocardial fibrosis (46). In high-hemodynamic-load HF model induced by Ang II., reducing Mitogen-Activated Protein Kinase (MAPK) could downregulate c-Myc, subsequently suppressing cardiac inflammation and fibrosis (50). Consistent with observations in load-induced HF models, myocardial c-Myc expression is also elevated in models of ischemic HF generated by ligation of the left anterior descending coronary artery. In this context, by inhibiting the Wnt/*β*-catenin pathway, the expression level of its downstream target c-Myc could be downregulated, thereby ameliorating inflammation and thereby attenuating cardiac fibrosis (59).

In activated MFs, c-Myc served as a critical signaling pathway for cell proliferation. Its upregulation mediated abnormal MFs proliferation, leading to excessive secretion of collagen and fibronectin, which results in aberrant accumulation of extracellular matrix and ultimately contributes to fibrosis in myocardial and other tissues (60–62). Beyond promoting MFs proliferation, c-Myc also induces fibroblasts to exert pathological effects on cardiomyocytes. A study identified a HF-specific cardiac fibroblasts population that is exclusively present in failing hearts. Interestingly, despite its high expression in this subpopulation, c-Myc did not drive cardiac fibrosis but instead exacerbates HF by regulating the chemokine CXCL1 to promote cardiomyocyte hypertrophy (63). In summary, c-Myc drives the pathogenesis of HF by promoting myocardial fibrosis, through its pleiotropic roles in the signaling networks of both cardiomyocytes and MFs.

### 3.3 c-Myc induces and sustains disturbances in energy metabolism

**Figure 3:**
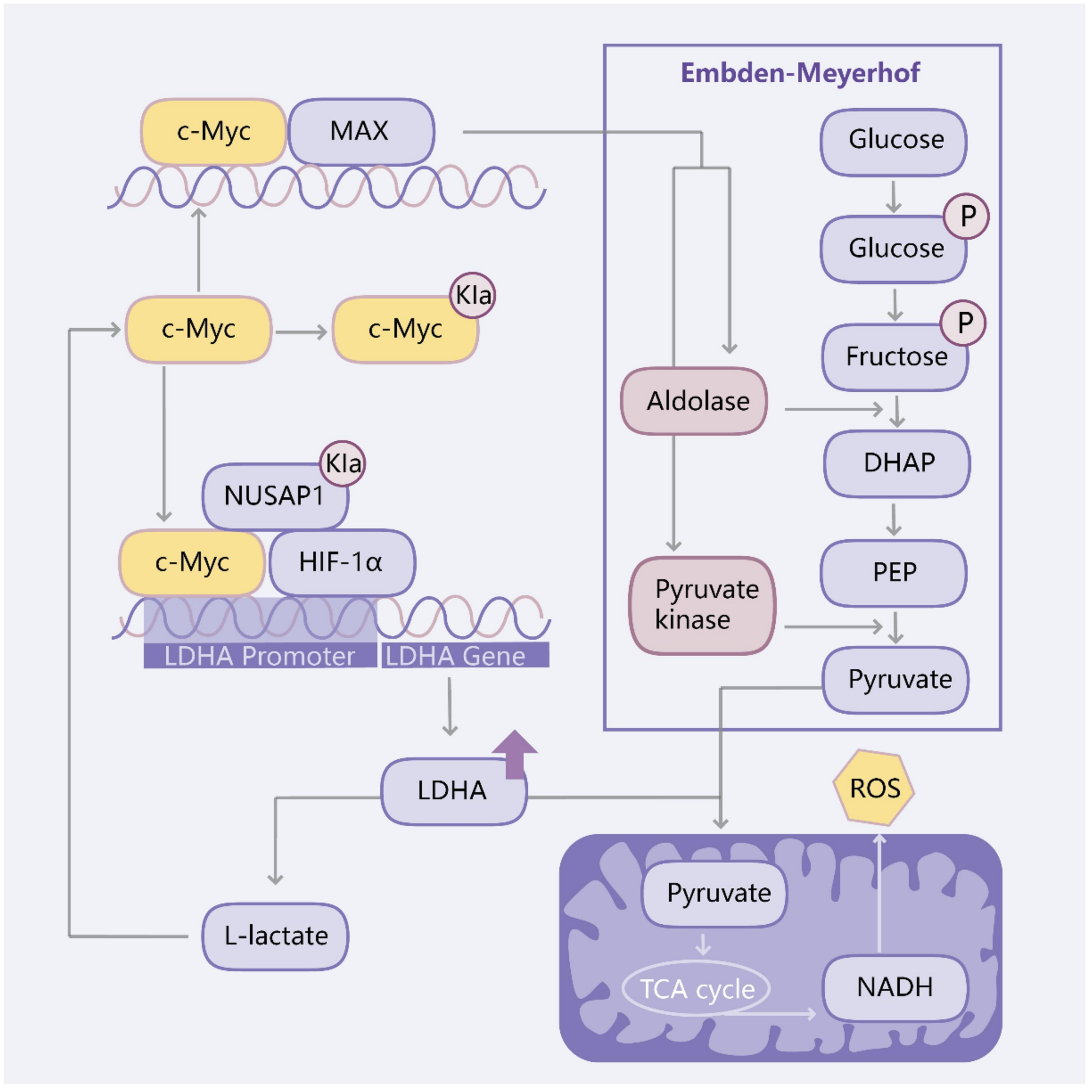
The role of c-Myc in energy metabolism in heart failure the c-Myc/MAX heterodimer fosters glycolysis by upregulating aldolase and pyruvate kinase expression. The c-Myc/HIF-1*α* transcriptional complex, assisted by lactylated NUSAP1, initiates high expression of LDHA, which expedites lactic acid production during glycolysis. In the resulting high-lactate environment, c-Myc may undergo lactylation modification, leading to enhanced activity, intracellular accumulation, and sustained oncogenic function. DHAP, dihydroxyacetone phosphate; HIF1-*α*, hypoxia-inducible factor 1-*α*; LDHA, lactate dehydrogenase A; MAX, MYC associated factor X; NADH, nicotinamide adenine dinucleotide; NUSAP1, nucleolar and spindle associated protein 1; PEP, phosphoenolpyruvate; ROS, reactive oxygen species; TCA, tricarboxylic acid cycle.

During HF induced by ischemia and other injurious factors, the energy metabolism of cardiomyocytes undergoes significant reprogramming. Under physiological conditions, the energy required for cardiac activity is predominantly derived from mitochondrial oxidative phosphorylation, with fatty acids as the primary substrate (64). However, the onset of myocardial ischemia triggers a metabolic shift toward glycolysis. In this adaptive response, various enzymes rapidly catalyze the breakdown of glucose into pyruvate, generating a limited yield of Adenosine Triphosphate (ATP) to sustain cardiac function (65). Under prolonged ischemic and hypoxic conditions, pyruvate derived from glycolysis is converted into lactate by lactate dehydrogenase A (LDHA) (66). The aberrant accumulation of lactate disrupts intracellular ion homeostasis and impairs mitochondrial structure and function, ultimately contributing to cardiomyocyte injury (67). Studies have suggested that c-Myc plays multifaceted roles in mediating this energy metabolic switch during ischemic HF (Figure 3).

On the one hand, c-Myc, as a key upstream transcription factor, involves in glycolysis and influences mitochondrial structure and function. Study has shown that c-Myc forms a heterodimeric complex with MAX, increasing transcription of several key glycolytic enzymes, including aldolase and pyruvate kinase and upregulating glycolytic metabolic flux (68). Pyruvate dehydrogenase kinase (PDK) is also a target gene of c-Myc. By transcriptionally upregulating PDK expression, c-Myc inhibits PDH activity, thereby suppressing the conversion of pyruvate to acetyl-CoA for entry into the tricarboxylic acid cycle (69). Additionally, c-Myc forms a transcriptional regulatory complex with hypoxia-inducible factor 1-*α* (HIF-1*α*) to initiate high expression of LDHA, promoting the conversion of pyruvate to lactate (70). Meanwhile, c-Myc participated in regulation of mitochondrial aerobic respiration. In adult mice cardiomyocytes with c-Myc overexpression or aberrant accumulation, decreased mitochondrial volume, reduced levels of mitochondrial complexes, and diminished oxidative phosphorylation metabolic activity were observed (48, 71).

On the other hand, in a high-lactate environment, c-Myc could undergo lactylation modification, resulting in increased activity and intracellular accumulation, allowing for its sustained function. In 2019, Zhang et al. firstly discovered lactylation modification on histone lysine residues activating chromatin gene transcription (72). In recent years, lactylation modifications have been identified in various proteins in myocardial tissue. Study showed that lactylation of histone H3 at lysine 18 enhances the transcriptional activity of atrial natriuretic peptide (ANP), thereby promoting cardiomyocyte hypertrophy and increased heart weight (73). Lactylation at the K166 and K728 sites of the non-histone protein trifunctional enzyme subunit alpha (HADHA) disrupted mitochondrial function by inhibiting HADHA activity and led to decreased cardiomyocyte contractility (74). The role of c-Myc lactylation in renal tubular endothelial cells during ischemia/reperfusion injury has been discovered. C-Myc lactylation, induced by heat shock protein A12A (HSPA12A), promoted the expression of proliferation-related genes by enhancing c-Myc nuclear localization, and leading to inducing the regeneration of injured renal tubular epithelial cells (32). However, whether this lactylation modification of c-Myc also occurs in cardiomyocytes, and and what specific role lactylated c-Myc plays in cardiomyocytes, remains to be further experimentally validated.

In summary, during the progression of ischemic HF, c-Myc acts as an upstream factor that orchestrates a metabolic shift toward glycolysis and disrupts mitochondrial structure and function. Meanwhile, lactate produced by glycolysis may induce lactylation of the c-Myc protein itself, potentially creating a feed-forward loop that sustains its biological effects. However, the precise mechanisms underlying this process require further investigation.

## 4 c-Myc in heart failure: protective effects

### 4.1 c-Myc is a potential contributor to cardiac regeneration and repair

In contrast to the detrimental effects discussed above, recent studies have shown that c-Myc also exerts beneficial effects under specific contexts, specifically by promoting cardiac regeneration and repair.

**Figure 4:**
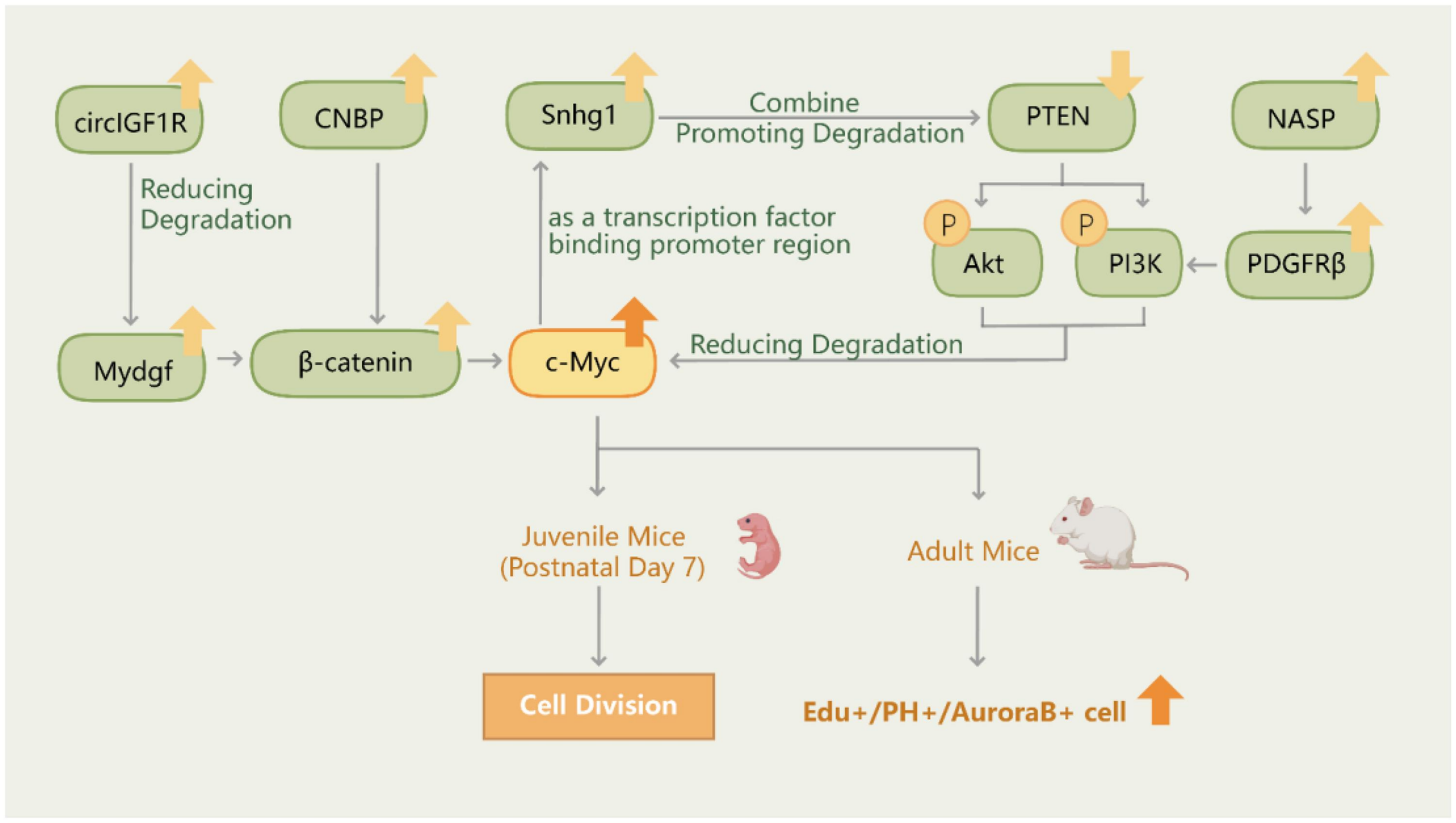
Role of c-Myc in cardiac regeneration and repair in heart failure overexpressed Snhg1 binds to PTEN and promotes its degradation, inhibiting c-Myc hydrolysis by enhancing Akt and PI3 K phosphorylation. Meanwhile, c-Myc, as a transcription factor, upregulates Snhg1 expression by binding to its promoter region, forming a positive feedback loop. This promotes cardiomyocyte division in neonatal mice and induces cell cycle re-entry in adult mice (evidenced by increased Edu^+^, PH3^+^, and AuroraB^+^ cells). Additionally, overexpression of circIGF1R, CNBP, Mydgf, and NASP upregulates c-Myc expression through the PI3 K/Akt and Wnt/*β*-catenin pathways, facilitating cell cycle re-entry. Akt, Ak strain transforming; AuroraB, aurora kinase B; circIGF1R, circular insulin-like growth factor 1 receptor; CNBP, cellular nucleic acid-binding protein; Mydgf, myeloid-derived growth factor; NASP, nuclear autoantigenic sperm protein; pdgfr*β*, platelet-derived growth factor receptor *β*; PH, pleckstrin homology domain; PI3K, phosphatidylinositol 3-kinase; PTEN, phosphatase and tensin homolog; Snhg1, small nucleolar RNA host gene 1.

Cardiomyocytes possess a markedly limited capacity for regeneration in adult mammals. Following injury, surviving cardiomyocytes primarily undergo hypertrophy and polyploidization to compensate for the increased workload, ultimately leading to ventricular remodeling and HF (75). Although the neonatal heart exhibits a transient phase of repair and regeneration after injury, this capacity declines rapidly shortly after birth (76). Consequently, in recent years, an emerging research focus has been on strategies to reactivate the self-renewal capacity of adult cardiomyocytes following cardiac injury or disease (77). Recent researches have indicated that the activation of the potential proliferation and regeneration of cardiomyocytes after cardiac injury shows an association with the c-Myc signaling pathway. It should be noted that current evidence of adult cardiomyocyte regeneration is largely based on indirect cell cycle markers, and more direct imaging evidence is needed for further validation (Figure 4).

Previous study showed that overexpression of c-Myc can induce cell cycle reentry, leading to nuclear replication and nuclear division, thereby increasing cardiomyocyte volume. However, no evidence of cardiomyocyte division and proliferation was observed (78). However, recent studies have uncovered phenomena that differ from those observed in the past. In 2021 a study in *Science* suggested that in neonatal mice (79), transient (6–12 days) and specific co-expression of Oct4, Sox2, Klf4, and c-Myc (collectively termed OSKM) extended the cardiac regeneration window. In adult mice, it induced limited mitotic activity (evidenced by increased Edu⁺, PH3⁺, and Aurora B⁺ cells), and reduced myocardial injury while improving cardiac function after myocardial infarction. However, overexpression of c-Myc alone did not replicate these effects.This suggests that overexpression of c-Myc could activate the regenerative potential of cardiomyocytes, but its efficacy necessitates co-operation with other reprogramming factors. Moreover, the timing of the intervention must be strictly controlled, as this study demonstrates that prolonged (21 days) OSKM expression led to cellular reprogramming and cardiac tumor formation. In addition to specific cooperating factors and precise timing, the level of c-Myc overexpression also exerts a profound influence on the biological effects. Another study suggested that (80), unlike the cardiomyocyte hypertrophy driven by strong overexpression of c-Myc in earlier research, moderate overexpression of c-Myc improved cardiac function and reduced scarring in apical resection-induced neonatal mice model. And it increased DNA synthesis in cardiomyocytes and increased heart weight, along with decreased individual cardiomyocyte size. Collectively, these phenotypes suggested that elevated DNA synthesis may associate with cardiomyocyte proliferation. However, this regenerative potential seems to be restricted to neonatal mice. No significant changes were observed in LAD ligation–induced adult mice model. In summary, the aforementioned studies indicate that c-Myc overexpression in cardiomyocytes may induce cardiac repair and proliferation to some extent. But the expression level, timing, and the co-activation of other reprogramming factors must be stringently controlled. And this repair and proliferative potential is more pronounced in neonatal cardiomyocytes compared to those from adult animals.

The upstream signaling pathways of c-Myc also implicated in the process of cardiomyocyte proliferation, as visually demonstrated by time-lapse imaging of juvenile mice cardiomyocytes. A study (81) showed that overexpression of the c-Myc upstream lncRNA small nucleolar RNA host gene 1 (Snhg1) induced cell cycle reentry in both neonatal and adult mice cardiomyocytes (evidenced by increased Edu+, PH3+, and Aurora B + cells), and increased the proportion of mononucleated cardiomyocytes. And cell division was observed through time-lapse imaging of primary cardiomyocytes labeled with fluorescent probes in juvenile mice (postnatal day 7). However, this study did not document divisional imaging in adult mice cardiomyocytes. The underlying mechanism involved a c-Myc/Snhg1 positive feedback loop. Snhg1 bound to Phosphatase and Tensin Homolog (PTEN) and promoted its degradation, leading to phosphorylation of Akt and PI3 K, thereby inhibiting the degradation of c-Myc. Concurrently, c-Myc, as a transcription factor, upregulated Snhg1 expression by binding to its promoter region, forming a reinforcing feedback loop ultimately. The Wnt/*β*-catenin pathway is an important upstream pathway of c-Myc and is activated by factors such as Krüppel-like factor 1 (KLF1) (82), cellular nucleic acid binding protein (CNBP) (83), and circular Insulin-like growth factor 1 receptor (circIGF1R) (84). Stabilized *β*-catenin can enter the nucleus and induce the expression of c-Myc, increasing expression of Ki-67 and pH3 and incorporation of EdU in cardiomyocytes. c-Myc is also regulated by various upstream factors, such as nuclear autoantigenic sperm protein (NASP) (85), which has been reported to upregulate c-Myc expression through activating the platelet-derived growth factor receptor (PDGFRB)/AKT pathway. In addition, the expression of c-Myc is also influenced by the myocardial microenvironment (86). A study showed that myeloid-derived growth factor (Mydgf), a paracrine protein mainly secreted by bone marrow-derived macrophages, can upregulate c-Myc expression through the PI3 K/Akt pathway (87). This subsequently leads to an extended window of cardiomyocyte proliferation and was associated with the promotion of cell cycle reentry in cardiomyocytes.

Based on the aforementioned studies, c-Myc-associated signaling pathways mediate cardiac proliferation might be promising. However, its expression level, timing, and the activation status of other reprogramming factors must all be subjected to stringent control. Moreover, in current researches, evidence for cardiomyocyte proliferation in adults primarily relies on the cell division marker proteins, cell volume, and organ weight. Direct validation through further time-lapse imaging evidence is still required.

### 4.2 The complex role of c-Myc in antitumor-therapy-induced cardiomyopathy

Anthracyclines such as Doxorubicin (DOX) are commonly used in cancer, but their clinical use is limited by cardiotoxicity (88). On the one hand, DOX -induced oxidative stress causes direct damage to cardiomyocytes (89). On the other hand, inflammation involving immune cells such as macrophages is also an important pathogenic mechanism. Local immune dysregulation leads to cardiomyocyte injury, ventricular remodeling, and cardiac fibrosis, ultimately resulting in HF (90). Studies have shown that c-Myc is involved in the pathogenesis of DOX-induced cardiomyopathy.

In macrophages, c-Myc and its upstream regulator, Scavenger Receptor Class A Member 1 (SR-A1), play a protective role against DOX-induced cardiomyopathy. The cardiac macrophage population primarily originates from two sources: monocyte-derived macrophages, which are recruited during injury and predominantly exert pro-inflammatory effects; and embryo-derived tissue-resident macrophages, which are primarily responsible for maintaining homeostasis and facilitating repair (91, 92). Study showed that upon DOX-induced cardiomyocyte injury, the SR-A1/c-Myc axis drives the proliferation of reparative macrophages attenuating cardiac inflammation, and consequently improving left ventricular systolic function (93).

In cardiomyocytes, the role of c-Myc during DOX-induced cardiomyopathy remains controversial. A study suggested that genistein (Gen) reduced DOX-induced cardiomyocyte apoptosis by inhibiting the phosphorylation of ERK1/2 and upregulating c-Myc protein levels (94). However, another study found that suppressing c-Myc expression partially reverses DOX-induced cardiomyocyte apoptosis and oxidative stress, reducing the severity of cardiac fibrosis (95). Neither of the studies examined the specific mechanisms by which c-Myc regulates cardiomyocyte apoptosis and oxidative stress, nor did they include *in vitro* cellular experiments. Therefore, while c-Myc and its upstream regulators may represent potential therapeutic targets for antitumor-therapy-induced cardiomyopathy, the precise mechanistic role of c-Myc in cardiomyocytes requires further experimental validation.

## 5 c-Myc is a critical driver of cardiac hypertrophy

Heart failure with preserved ejection fraction (HFpEF) is a subtype of HF, accounting for over one-half of all HF cases (96). HFpEF and heart failure with reduced ejection fraction (HFrEF) are mechanistically distinct (97). The aforementioned studies on c-Myc in HF have been primarily conducted in patients or animal models with HFrEF. In contrast, the role of c-Myc in HFpEF remains insufficiently supported by evidence, with only some studies suggesting its potential involvement in the pathogenesis of HFpEF.

HFpEF is closely associated with obesity, type 2 diabetes, and metabolic syndrome, with over 80% of HFpEF patients being overweight or obese (98). Insulin resistance is one of the core mechanisms of HFpEF, impeding glucose uptake by cardiac cells and disrupting anti-inflammatory signals (99). Bioinformatics analysis and *in vitro* experimental validation have identified c-Myc as one of the hub genes in diabetic cardiomyopathy, with its mRNA levels upregulated in mouse models of diabetic cardiomyopathy (100). c-Myc inhibition increased GLP-1 level and ameliorated high-fat diet-induced obesity and insulin resistance (101). These findings suggest that c-Myc may be involved in HFpEF by modulating glucose metabolism, though this hypothesis awaits further investigation in HFpEF animal models or patient cohorts.

Chronic inflammation induced by metabolic abnormalities is another important pathological mechanism of HFpEF. Metabolic abnormalities lead to phenotypic changes in epicardial adipose tissue, increasing the secretion of chemokines and promoting immune cell infiltration (102). Meanwhile, Insulin resistance activates the nuclear factor *κ*-B (NF-*κ*B) pathway in immune cells, upregulating inflammatory cytokines and establishing a state of “metabolic inflammation” (98). Cardiome-directed network analysis (CDNA) based on heart tissues from HFpEF rats (the obese and hypertensive ZSF1 rat model) identified c-Myc as a key bottleneck in the overall network topology involved in metabolic inflammation in HFpEF (103). Although direct evidence remains limited, studies have suggested that c-Myc may regulate the inflammatory process in HFpEF through multiple pathways. In hepatocellular carcinoma, c-Myc has been shown to increase the expression of the chemokine vascular cell adhesion molecule 1 (VCAM1), thereby promoting cell migration and recruitment (104). In inflammatory macrophages, c-Myc regulated glucose metabolism and increased the expression of tumor necrosis factor (TNF)-*α* and interleukin (IL)-6, thereby aggravating inflammation (105). In addition, c-Myc was predicted by Ingenuity Pathway Analysis (IPA) as an upstream hub gene for fibroblast activation in diabetic model mice (106). The mechanism may involve c-Myc upregulating IL-10 and transforming growth factor (TGF)-β (107), which in turn induce fibroblast activation and extracellular matrix remodeling through osteopontin, ultimately leading to cardiac fibrosis and diastolic dysfunction (102). Collectively, these findings suggest that c-Myc may serve as a bridging molecule linking metabolic dysregulation, inflammation, and fibrosis in HFpEF, but further experimental validation is warranted.

It should be noted that coronary microvascular endothelial dysfunction is also one of the important pathological features of HFpEF (108). However, unlike the aforementioned role of c-Myc in exacerbating metabolic dysregulation and inflammation, c-Myc appears to exert a protective effect against vascular endothelial dysfunction. A study has shown that c-Myc conditional knockout mice develop endothelial dysfunction, whereas overexpression of endothelial c-Myc attenuated diet-induced endothelial injury (109). In diabetic models, c-Myc knockdown suppresses the endothelial protective effects of resveratrol (110) and acidic fibroblast growth factor (111), leading to aggravated endothelial.

**Figure 5:**
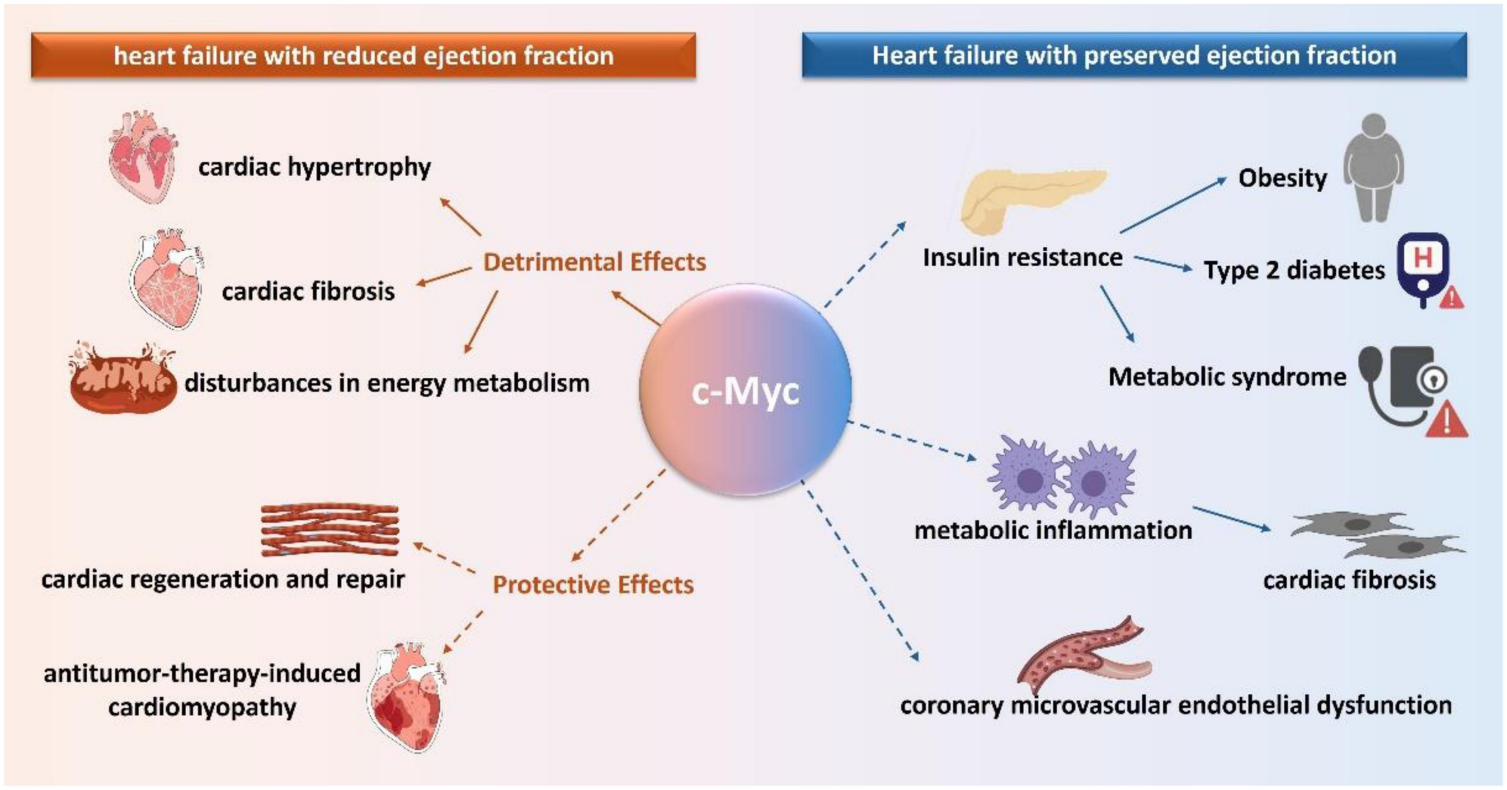
Differential roles of C–Myc in HFrEF and HFpEF. In HFrEF, c-Myc promotes cardiac remodeling, fibrosis, and metabolic dysregulation, while potentially suppressing HFrEF through cardiac repair and alleviation of drug-induced cardiomyopathy. In HFpEF, c-Myc might drive insulin resistance, metabolic inflammation, and fibrosis, thereby promoting HFpEF progression, while potentially counteracting HFpEF by protecting against coronary microvascular endothelial dysfunction.

In summary, although direct evidence for the role of c-Myc in HFpEF remains insufficient, available clues suggest a trend toward dual effects: promoting metabolic dysregulation, inflammation, and fibrosis, while potentially protecting endothelial function. This complexity indicates that the effects of c-Myc are highly dependent on cell type and pathological context, and systematic studies across different cell types in HFpEF models are needed to clarify its pathophysiological significance (Figure 5).

## 6 Targeting c-Myc: therapeutic strategies

Because of its nuclear localization and lack of deep protein pockets, previous studies have considered c-Myc difficult to drug (112). In recent years, there has been a paradigm shift in therapeutic approaches targeting c-Myc, and a growing number of direct and indirect therapeutic approaches targeting c-Myc are emerging. According to some academic perspectives, it is a case of “when” c-Myc will be a drugged clinical target, rather than “if” (113). However, given the dual role of c-Myc in diseases, its clinical application across different diseases and stages requires extensive experimental investigation.

### 6.1 c-Myc inhibitor

In most cases, the overactivity or overexpression of c-Myc is considered deleterious, driving uncontrolled proliferation, aberrant differentiation, and metabolic reprogramming, leading to pathological outcomes such as tumorigenesis and ventricular remodeling. Thus, the development of c-Myc inhibitors holds considerable potential.

Extensive research has been conducted on c-Myc-targeted oligonucleotide therapeutics based on small interfering RNA (siRNA) and short hairpin RNA (shRNA). In 2008, Wang et al. first developed an shRNA targeting c-Myc, which was delivered via a lentiviral-based expression vector into human tumor cells and normal human cells, resulting in stable reduction of c-Myc (114). Subsequent studies demonstrated that transfection of c-Myc-targeting siRNA into neonatal rat cardiomyocytes or H9C2 cells using a liposome-based delivery system effectively reduced both transcriptional and protein levels of c-Myc, thereby inhibiting or reversing drug-induced cardiomyocyte hypertrophy (46, 50). In recent years, several studies have explored the *in vivo* application of c-Myc-targeting siRNA in the oncology field. A lipoplex based on pre-compression of c-Myc-targeting siRNA was developed. By intranasal delivery, this complex effectively suppressed c-Myc expression in intracranial glioblastoma and extended survival in mice (115). A c-Myc-targeting siRNA delivered by lipid-based nanoparticles was developed and advanced to a Phase I clinical trial for the treatment of multiple tumors; however, the trial was discontinued due to insufficient gene silencing (113). In summary, while c-Myc-targeting siRNA has been validated *in vitro* for ameliorating cardiomyocyte hypertrophy, and the *in vivo* effects of this siRNA have been preliminarily explored in oncology, research on c-Myc siRNA specifically for HF remains largely unexplored.

Small-molecule compounds designed to disrupt protein-protein interactions also represent a promising direction for the development of c-Myc inhibitors. The mini-protein Omomyc, developed by Laura Soucek and her team, stands as a landmark achievement in the application of c-Myc inhibitors in the field of oncology. Omomyc dimerizes with MAX, occupying the DNA with transcriptionally inactive complexes that interfere with transactivation of c-Myc (116, 117). In both cellular and animal experiments, Omomyc demonstrated significant antitumor properties across a variety of tumor types (118). In clinical trial, c-Myc showed safety and positive signs of target engagement and drug activity (119). Currently, Omomyc is undergoing Phase Ib and Phase II clinical trials (113).

**Figure 6:**
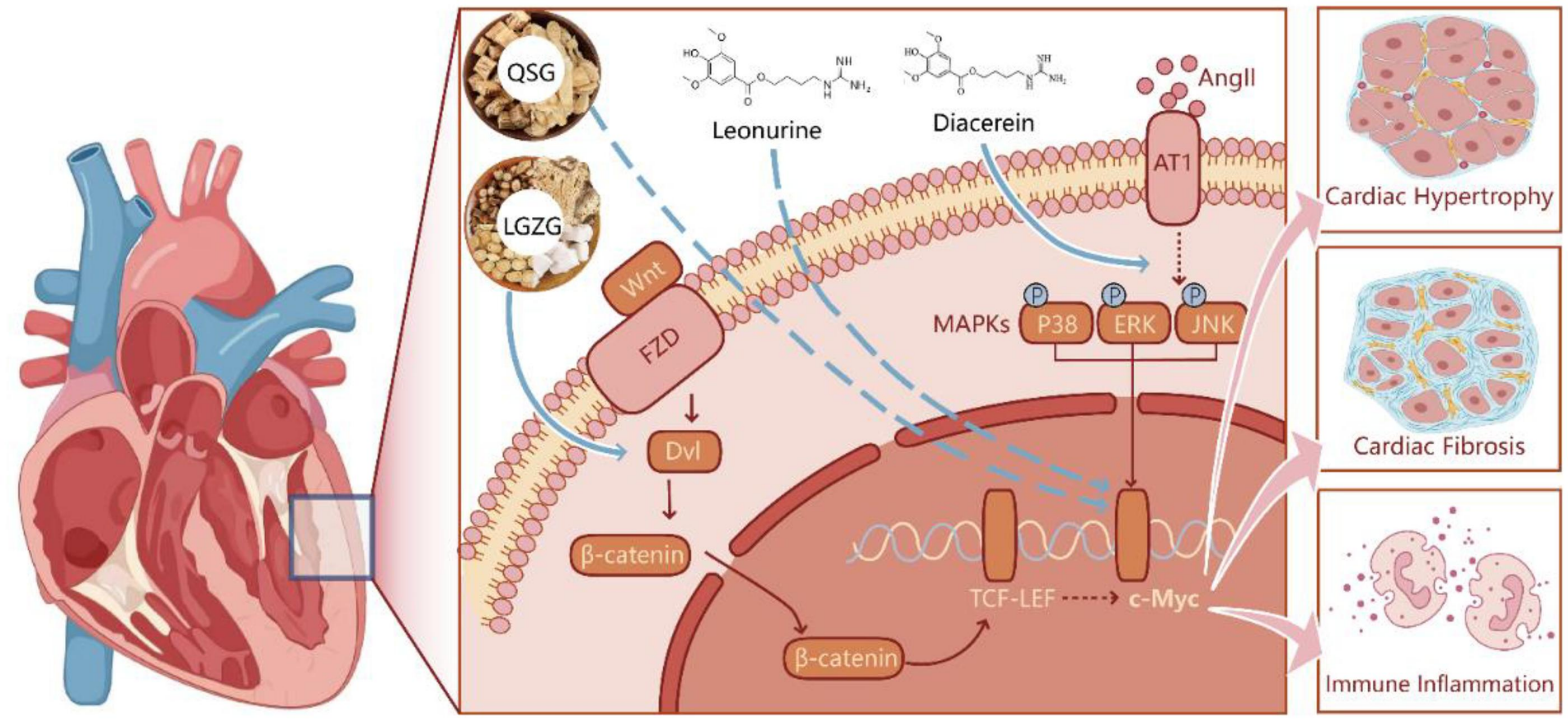
Mechanisms of Action of Traditional Chinese Medicine in Inhibiting c-Myc Linggui Zhugan Decoction, Qishen Granules, and monomers Leonurine or Diacerein downregulate c-Myc by inhibiting the Wnt/*β*-catenin and MAPK pathways, and consequently alleviate cardiac hypertrophy, fibrosis, and inflammation in heart failure. AngII, angiotensin II; AT1, angiotensin ii receptor type 1; EPK, extracellular signal-regulated kinase; FZD, frizzled; Dvl, Dishevelled; JNK, c-Jun N-Terminal Kinase; LGZG, Linggui Zhugan Decoction; MAPKs, mitogen-activated protein kinases; P38, p38 mitogen-activated protein kinase; QSG, qishen granule; TCF-LEF, T cell factor - lymphoid enhancer-binding factor.

Traditional Chinese medicine (TCM) has demonstrated unique advantages in the clinical management of HF, supported by extensive practical experience. Recent studies have indicated that TCM interventions exhibit significant inhibitory effects on c-Myc in the context of HF treatment, suggesting a promising new direction for developing therapeutic strategies targeting c-Myc. Study showed that TCM compound Linggui Zhugan inhibited the Wnt/*β*-catenin signaling pathway, reduced c-Myc levels, delayed cardiomyocyte hypertrophy and fibrosis, and improved cardiac function in post-myocardial infarction heart failure mouse (59). The proprietary Chinese medicine Qishen Granule ameliorates HF by reducing c-Myc levels, decreasing reactive oxygen species (ROS) release, attenuating inflammation, and ultimately enhancing cardiac function (120). In addition to TCM compound, ingredients extracted from TCM herbs also possess c-Myc inhibitory effects. Diacerain extracted from natural plants was shown to mitigate cardiac fibrosis and hypertrophy. This effect was mediated through the inhibition of the MAPKs/c-Myc pathway, leading to a reduction in inflammatory responses both *in vivo* and in cultured cardiomyocytes (50). In an overload-induced cardiac hypertrophy rat model, leonurine, extracted from natural plants, suppressed c-Myc transcription, contributing to the reduction of cardiac hypertrophy, edema, and inflammatory cell infiltration, consequently improving cardiac function (121) (Figure 6).

### 6.2 c-Myc activator

In certain specific circumstances, the activation of c-Myc could be beneficial, primarily in diseases that require the promotion of cell proliferation and tissue regeneration, such as post-ischemic cardiac repair (97–100), pancreatic islet cell proliferation in diabetes (122), and neural cell repair (123–125). However, studies on the activation or overexpression of c-Myc have not yet been conducted in human clinical trials, primarily due to limitations in the precision of intervention strategies. In ischemic HF, it is crucial to achieve localized and specific activation of c-Myc in cardiac tissue. This requires precise regulation without adversely affecting other organs, thereby avoiding the severe side effects (such as tumorigenesis). Moreover, a critical challenge remains in precisely regulating c-Myc to balance its role in cell proliferation against the risk of hypertrophy. Given its bidirectional regulatory effects and holistic mechanisms, TCM presents a promising approach for the precise modulation of c-Myc, warranting further in-depth exploration.

## 7 Conclusion and future perspectives

c-Myc is widely involved in the pathological process of the occurrence and development of HFrEF, but its function is highly diverse. Under normal circumstances, the heart with HFrEF exhibits high expression of c-Myc and its related signaling pathways, contributing to the pathological progression of HF through cardiac hypertrophy, cardiac fibrosis, and metabolic reprogramming. However, under specific limiting conditions, the upregulation of c-Myc might hold the potential to drive cardiomyocytes to reinitiate self-renewal, exerting a protective effect on cardiac function. This bidirectional and complex role of c-Myc may be attributed to its expression level, timing of activation, and the involvement of other related factors. Strong and prolonged upregulation of c-Myc in injured cardiomyocytes can induce pathological processes such as cardiac hypertrophy, fibrosis, and metabolic reprogramming. In contrast, transient and moderate upregulation of c-Myc, accompanied by the activation of other reprogramming factors, may promote cardiac repair to a certain extent. In summary, further studies are needed to elucidate the distinct biological roles of c-Myc at different expression levels across various disease stages, etiologies of heart failure, and cell types.

Research on the role of c-Myc in HFpEF remains insufficient. Available evidence suggests that its effects differ from those observed in HFrEF and also exhibit a bidirectional trend: c-Myc may participate in metabolic dysregulation, promote inflammation and fibrosis, while potentially exerting a protective effect on vascular endothelium. Therefore, systematic studies across different cell types in HFpEF models are needed in the future to elucidate its pathophysiological significance.

Current research on c-Myc is primarily at the preclinical stage. Looking forward, in order to facilitate the clinical translation of c-Myc as a potential therapeutic target for HF, several critical challenges and promising directions should be addressed:

**Precise Targeting:** As a proto-oncogene, systemic modulation of c-Myc (particularly its activation or overexpression) could increase the risk of several side effects such as cancer. Moreover, c-Myc could exert distinct effects in different cells, such as cardiomyocytes and myofibroblasts as previously mentioned. Therefore, achieving cell-type-specific regulation of c-Myc represents a primary current challenge. A key focus of future research lies in developing advanced drug delivery systems, such as nanoparticles or cardiac-specific viral vectors, to precisely deliver c-Myc-modulating therapeutics to specific cellular subpopulations. This approach will enable localized modulation, minimize off-target effects, and reduce carcinogenic risks.
**Optimal Expression and Timing:** The biological effects of c-Myc are significantly influenced by its expression level and the timing of activation. During the early phase of myocardial ischemia, timely induction of moderate c-Myc overexpression may hold potential for restoring cardiomyocyte repair mechanisms. Conversely, in the chronic stage of HF, suppression of c-Myc may delay and ameliorate ventricular remodeling. Therefore, more systematic and large-scale studies are required to clarify the functional differences of c-Myc at different stages of HF progression and under different expression levels, thereby determining the optimal “expression window” and “therapeutic time window” for c-Myc-targeted intervention.
**Integrated Mechanisms:** Current understanding of the mechanistic network through which c-Myc operates in HF remains incomplete. The transcriptional regulation of the c-Myc protein, its post-translational modifications, its related signal transduction pathways, and the crosstalk between these pathways all represent potential points for therapeutic intervention. Each of these aspects warrants extensive experimental investigation and validation.
**TCM and Holistic Modulation**: The role of c-Myc in HF is characterized by bidirectionality and complexity. Studies have suggested that TCM interventions hold potential value in modulating c-Myc. However, the current evidence is preliminary, and more high-quality studies are needed, and several challenges remain to be addressed, including the identification of components in TCM formulas and the reproducibility of research findings.
In summary, although targeting c-Myc for HF therapy is challenging, it holds certain promise. Advances in future research on c-Myc modulation are expected to provide novel strategic insights for the treatment of HF.
